# Optimizing Deep Learning Models for Climate-Related Natural Disaster Detection from UAV Images and Remote Sensing Data

**DOI:** 10.3390/jimaging11020032

**Published:** 2025-01-24

**Authors:** Kim VanExel, Samendra Sherchan, Siyan Liu

**Affiliations:** 1 Bioenvironmental Sciences Department, Morgan State University, Baltimore, MD 21251, USA; 2 Center for Climate Change & Health, Morgan State University, Baltimore, MD 21251, USA; samendra.sherchan@morgan.edu; 3 Oak Ridge National Laboratory, Oak Ridge, TN 37830, USA

**Keywords:** AI, climate change, flooding, desertification, neural networks, CNN, transfer learning, UAVs, remote sensing, satellite

## Abstract

This research study utilized artificial intelligence (AI) to detect natural disasters from aerial images. Flooding and desertification were two natural disasters taken into consideration. The Climate Change Dataset was created by compiling various open-access data sources. This dataset contains 6334 aerial images from UAV (unmanned aerial vehicles) images and satellite images. The Climate Change Dataset was then used to train Deep Learning (DL) models to identify natural disasters. Four different Machine Learning (ML) models were used: convolutional neural network (CNN), DenseNet201, VGG16, and ResNet50. These ML models were trained on our Climate Change Dataset so that their performance could be compared. DenseNet201 was chosen for optimization. All four ML models performed well. DenseNet201 and ResNet50 achieved the highest testing accuracies of 99.37% and 99.21%, respectively. This research project demonstrates the potential of AI to address environmental challenges, such as climate change-related natural disasters. This study’s approach is novel by creating a new dataset, optimizing an ML model, cross-validating, and presenting desertification as one of our natural disasters for DL detection. Three categories were used (Flooded, Desert, Neither). Our study relates to AI for Climate Change and Environmental Sustainability. Drone emergency response would be a practical application for our research project.

## 1. Introduction

In this Section, we will first introduce the topic of climate change, the impact of climate change and then climate change-related natural disasters. We will then review the relevant literature on related AI studies. We will discuss the open-access data sources used and relevant AI studies utilizing this data site. At the end of this Section, we will present the significance and importance of this research study.

### 1.1. Introduction on Climate Change

According to the 2021 Intergovernmental Panel on Climate Change (IPCC) report, every continent worldwide has exhibited a rise in average temperatures due to anthropogenic climate change [1,2]. Heat waves, which developed before the Industrial Revolution with a probability of 1 in 10, will now occur 2.8 times more often and be even hotter than before [2]. Globally, thirty-seven percent of mortalities due to overheating were caused by global warming [3]. Climate change has been made more apparent through the intensity of extreme weather events such as heatwaves, hurricanes, heavy precipitation, flooding, etc. [1].

Flooding alone has led to over USD 610 billion in damages [1,4]. Research studies have demonstrated that the rising intensity of floods has been driven by climate change [1,5,6,7,8,9]. This project addresses the detection of extreme weather, natural disasters, and the resulting environmental degradation of land by utilizing AI, ML, and DL techniques.

Desertification can be described as the degradation of the land in arid, dry sub-humid, semi-arid regions due to climate change and other anthropogenic activities [10,11]. Global warming accelerates desertification [10,12]. The variations in weather patterns caused by climate change exacerbate desertification [13]. Approximately 25% of the land surface worldwide is being impacted by desertification [10,11,14]. Research modeling has predicted that the (moderate–very high) risk of desertification will rise by twenty-three percent before 2100 in their high greenhouse gas emissions scenario [10].

### 1.2. Current Methods on Impact of Climate Change

Current methods to study the impact of climate change were explored [1,2,10]. Daily maximum temperature measurements, heatwave duration, and heatwave frequency have been utilized to measure and predict rising global average temperatures [1]. Clarke et. al. utilized numerous sources to measure the negative impact from climate change-related flooding events including insured damage, recorded deaths, precipitation, climate model simulations, etc. Data from the IPCC report, which is a valuable resource regarding climate change, were included in their article [2]. Huang et. al. utilized the global desertification vulnerability index to describe and predict the climate change impacts from desertification [10]. Carbon dioxide emissions, gross domestic product, population density, and population growth were used to calculate the human activity index [10]. Temperature anomaly, aridity index, and leaf area measurements were utilized to calculate the climate environment index. Together, the human activity index and the climate environment index determined the global desertification vulnerability index [10]. The impact of climate change and desertification was indicated by this index. The current methodology in AI to study the impact of climate change will be discussed later in Section 1.3.

Before our research study, desertification was not included as a natural disaster for DL detection. Due to the impending threat of desertification and its ramifications, our project includes a desertification category among the total categories considered for DL image classification. Our novel dataset, the Climate Change Dataset, includes 1931 images within the desertification category.

### 1.3. Related AI Research Studies

Other research studies have delved into AI detection of such extreme weather events and natural disasters as flooding, etc. [15,16,17,18,19]. In a study by Daniel Hernández et al., their research group developed an AI pipeline to detect natural disasters such as flooding [15]. Their project enables efficient processing of UAV images. Their study was limited by the need for manual labeling of test images. Albandari Alsumayt et. al. proposed the Flood Detection Secure System (FDSS), which utilized drones for image classification of flooding events while keeping the data secure [16]. Their research study furthers progress in cybersecurity efforts to secure and decentralize natural disaster monitoring by UAVs. At that time, the research article was limited by not including the results from the implementation of their proposal. Naili Suri Intizhami et al. present their flood area dataset containing images of flooding in Indonesia. Their dataset also includes color-labeled annotation for computer vision research on flooding identification [17]. Their research study enables the potential for real-time natural disaster detection from social media images. They offer potentially valuable research for computer vision studies. Their project is limited by the inconsistent image quality of videos on social media. Fatma S. Alrayes et al. developed their own AISCC-DE2MS technique for emergency disaster monitoring by drones, which includes encryption and image classification functionality [18]. Their project furthers cybersecurity efforts to secure drone communication during natural disaster detection. Their study is limited by the dataset used to test their novel technique. R. Karanjit et al. introduced their novel dataset, Flood Image (FloodIMG), in combination with DL techniques to classify flooding events [19]. Their dataset was assembled using the Internet of Things (IoT) application programming interfaces they developed. Their study’s natural disaster detection was limited to flooding. Our research project seeks to accurately detect natural disasters such as flooding and desertification. Desertification has become an increasingly critical matter, which has rarely been addressed previously in deep learning AI disaster detection projects from images.

Recent research studies have explored how AI techniques have been applied to the field of agriculture [20,21,22]. Malik et. al. studied the impact of climate stress on agriculture in Jammu, Kashmir, and Ladakh [20]. Their research demonstrated the current methodology for the impact of climate change on agriculture. Malik et. al. utilized two government data sources containing data such as maximum/minimum temperatures [20]. Estimates on agricultural growth were made using the Ricardian method [23]. An LSTM (long short-term memory)-based neural network was used to predict future climate variabilities [20]. Their results showed that climate change’s effect on climate variables had a negative impact on agricultural growth [20]. A resulting decrease in landholdings, also, has a negative impact on agricultural growth. Seelwal et al. reviewed how DL was utilized for rice disease diagnosis [21]. Their review selected 69 studies related to rice disease and DL detection. The most common machine learning model deployed was the CNN. Alkanan and Gulzar studied AI corn seed disease classification [22]. They furthered AI research in the field of agriculture. Their study utilized MobileNetV2 for image classification.

### 1.4. AI in Aerial Imagery

Tools to accurately detect and predict natural disasters are needed to prevent and lower the resulting property damage along with the number of resulting mortalities. Significant progress has been made in recent years in AI detection of natural disasters. These methods often use remote sensing, satellite imagery, and/or unmanned aerial vehicle (UAV) images in their training dataset [15,16,18,19]. Surveillance tactics are required to notify the public in adequate time of impending danger from extreme weather events.

For decades now, satellites have been monitoring and providing beneficial observations of land and sea conditions [24] at moderate resolution and at coarse resolution. In recent years, research studies have been able to utilize high-resolution satellite images of less than five meters [25]. UAV imagery has also begun to play an increasing role in AI research studies in the environmental science/agriculture fields [26,27,28].

Dilmurat et al. monitored changes in crops through UAV LiDAR and hyperspectral imaging to accurately forecast maize yield with their H20 Automated Machine Learning framework [26]. Damini Raniga et al. presented a workflow of AI detection and monitoring of the health condition of the delicate vegetation in the East Antarctic’s protected area by utilizing non-invasive UAV images [27]. Andrea Santangeli et al. were able to effectively combine UAV thermal imaging with AI detection to precisely identify bird nests lying on agricultural fields with the intent that their tractors should avoid the ground-level bird nests [28].

UAVs, also known as drones, have been utilized for flood image classification/detection purposes, as well [15,16,18]. For instance, Daniel Hernández et al. proposed an AI-based pipeline, taking drone images of floods as input, extracting key features, reducing the complexity of the feature data, grouping unlabeled images by similarity, and then sending prototypes of the clusters for manual labeling of drone images by natural disaster first responders [15].

UAV imagery has a higher spatial resolution than satellite imagery and can often offer greater accuracy in image classification [29]. UAV images have many advantages besides high-resolution imaging including the ease of transportation and deployment [25]. Drones can be used during cloudy weather conditions and still produce quality aerial images [30]. UAVs used for Low Altitude Sensing Systems (LARS) offer enhanced flexibility [30]. Drones cannot yet rival the amount of spatial area that satellites are able to cover but UAV data could potentially complement and augment satellite data [25].

Several research studies have effectively combined both UAV and satellite remote sensing imaging for classification/detection purposes [19,25,31]. Dash et al. performed a controlled study on the effects of herbicide on *P. radiata* through analysis of both UAV and satellite remote sensing imaging [25]. Karanjit et al. compiled images of flooding from a variety of sources: Google Search, Twitter, DOT traffic cameras, GitHub, USGS, etc., with huge variations in resolution size for AI-driven flood detection [19]. Marx et al. utilized Landsat satellite data in combination with UAV images of remote areas uncaptured by high-resolution satellite imagery to document deforestation and reforestation events [31].

### 1.5. AI Research Utilizing Our Data Source

An open-access data site, Kaggle, has become well-known and utilized in research studies [32,33,34,35,36,37]. Kaggle datasets containing medical imaging have been used in disease detection [32,33,34,36,37]. Hassan et al. utilized three benchmark datasets to present their novel multi-stage deep neural network architecture for the detection of Alzheimer’s disease [32]. Land-use/land-categorization articles have benefited from Kaggle datasets, as well [38,39]. Kwenda et al. introduce a hybrid approach combining deep neural networks and ML algorithms trained on the deep globe challenge dataset for the purposes of image segmentation of satellite images into forest vs. non-forest regions [38]. Natural Learning Processing (NLP) research has also made use of Kaggle datasets [40]. Kaggle datasets can be found in cybersecurity research [41]. Amnah Albin Ahmed et al. utilized ensemble and DL models trained on the Android Ransomware Detection dataset for the detection of such cyberattacks [41]. Kaggle competitions have even been studied and analyzed in research journals [35,42]. Souhaib Ben Taieb and Rob J. Hyndman analyzed the approach used by their team in the Load Forecasting track of the Kaggle Global Energy Forecasting Competition [42].

This research focuses on AI-based flood detection. Previous studies have utilized Kaggle datasets for flood detection [17,19]. Naili Suri Intizhami et al. posted one such open-access dataset [17]. Intizhami et al. utilized images posted on social media of flooding in South Sulawesi Indonesia [17]. With these images, Intizhami et al. built their own dataset color annotated with six different classes for use by ML/DL models for image segmentation purposes [17]. This project trained ML/DL models on our Climate Change Dataset compiled from various related Kaggle datasets.

### 1.6. Purpose of Our Research Study

The aim of this project was (1) to harness the capabilities of AI, ML, DL, and Data Science to tackle complex environmental issues; (2) to utilize existing aerial images to build AI/ML models to detect natural disasters such as flooding or desertification; and (3) to demonstrate the ability of AI/ML to be used for humanitarian efforts such as research on climate change crisis problems. To achieve these goals a dataset was compiled of aerial images, machine learning models were built, transfer learning was utilized, and one of our top-performing ML models was optimized.

We hypothesize that DL image classification techniques can detect climate change-related natural disasters, such as flooding and desertification, based on aerial images of the given area with an accuracy surpassing seventy percent. The significance of this study is due to the increase in the incidence and intensity of climate change-related extreme weather events and natural disasters. There is a call for a more robust response to natural disasters. AI detection of natural disasters could be utilized by drone emergency response. This study contributes to the literature by (1) offering a novel dataset with over 6 K images, (2) providing DL model optimization techniques, (3) including desertification as a natural disaster for DL detection purposes, (4) utilizing cross-validation dataset methods, (5) comparing ML model performance for our dataset, and (5) offering natural disaster detection methods for drone emergency response purposes.

## 2. Materials and Methods

In this Section, we will first introduce the dataset we collected for this work, followed by the data pre-processing, model building, and selections, and then experiment setup and evaluation metrics.

### 2.1. Compiling the Climate Change Dataset

We will now explain how our dataset was assembled. Over 6.3 K aerial images from unmanned aerial vehicles and satellite images were collected and used to form the compiled Climate Change Dataset, totaling 6334 images. A colleague in AI research recommended that we try the open-access data site, Kaggle [43]. Multiple datasets from Kaggle [43] were utilized including Louisiana Flood 2016 [44], FDL_UAV_flood areas [45], Cyclone Wildfire Flood Earthquake Database [46], Satellite Image Classification [47], Disaster Dataset [48], Aerial Landscape Images [49], Aerial Images of Cities [50], and Forest Aerial Images for Segmentation [51].

- The Louisiana Flood 2016 [44] dataset:
  ○ contained aerial images from the historic flooding that occurred in Southern Louisiana in 2016. For each image taken during the flood, there was a corresponding image before/after the flood.
  ○ image size: 512 × 360 pixels.
- The FDL_UAV_flood areas [45] dataset:
  ○ contained aerial images of Houston, TX from Hurricane Harvey. The dataset contains both flooded and unflooded images.
  ○ the image dimensions were approximately 3 K × 4 K pixels.
- The Cyclone Wildfire Flood Earthquake Database [46]:
  ○ contained videos and images from various natural disasters. We selected images from the Flood folder. These images were obtained from a Google search on each natural disaster included in the dataset.
  ○ the images were of variable sizes.
- The Satellite Image Classification [47] dataset:
  ○ was created from sensors and Google Map snapshots.
  ○ images size: 256 × 256 pixels.
- Disaster Dataset [48]:
  ○ contains images from numerous natural disasters.
  ○ the images were resized to 224 × 224 pixels.
- Aerial Landscape Images [49]:
  ○ curated dataset of aerial landscapes from 2 publicly available data sources, AID and NWPU-Resisc45 [52,53]. This dataset was intended for the field of computer vision. Of their 15 total categories, we selected the Desert category.
  ○ image size: 256 × 256 pixels.
- Aerial Images of Cities [50]:
  ○ urban aerial landscape images compiled from AID [52] and NWPU-Resisc45 [53] datasets.
  ○ images of size: 256 × 256 pixels.
- Forest Aerial Images for Segmentation [51]:
  ○ satellite images of forest land cover. Dataset was obtained from Land Cover Classification Track in DeepGlobe Challenge [51].
  ○ images resized to 256 × 256 pixels.

The Climate Change Dataset contains 3 categories: Flooded, Desert, and Neither. Both flooding and desertification are climate change-related natural disasters. The Neither category represents those images that are neither flooding nor desertification. These 8 datasets were found by a search through the Kaggle data site. The datasets with the most copious and relevant images were selected. Preference was given to more recently posted datasets. Unless specified above, the datasets listed above mentioned no further preprocessing steps. Our own preprocessing steps are mentioned below in Section 2.2.

The Flooded category contains over 2 K aerial images of flooded residential areas (2338 images). Pertinent images for the Flooded category were selected from Louisiana Flood 2016 [44], FDL_UAV_flooded areas [45], Cyclone Wildfire Flood Earthquake Database [46], and the Disaster Dataset (subset comprehensive disaster dataset/water disaster and the disaster dataset final/flood subset) [48]. These datasets were found by a search of flooded images from the same open-access data site. Preference was given to larger datasets.

The Desert category contains approximately 2 K aerial images of desert areas (1931 images). Relevant images for the Desert category were selected from Satellite Image Classification (Desert subset) [47] and Aerial Landscape Images (Desert subset) [49]. The Satellite Image Classification dataset [47] was recommended by a colleague in AI research. The Aerial Landscape Images dataset [49] was found through a search on Kaggle for desert images. Each dataset was chosen for its numerous images related to the desertification category.

The Neither category contains over 2 K aerial images of non-flooded residential areas and non-flooded forested areas (2065 images). Table 1 shows the image count for each data source. Qualifying images were selected from Louisiana Flood 2016 [44], FDL_UAV_flooded areas [45], Aerial images of Cities (Residential subset) [50], Disasters Dataset (neutral images subset) [48], and the Forest Aerial Images for Segmentation [51]. Images of unflooded residential images were needed. The Louisiana Flood 2016 [44], FDL_UAV_flooded areas [45], Aerial images of Cities [50], and the Disasters Dataset [48] each contained a subset of unflooded residential images. The Aerial Images of Cities dataset [50] was found via a search on Kaggle for residential areas. The Forest Aerial Images for Segmentation dataset [51] contained images of non-flooded forest areas. This dataset was found by a search on the same open-access data site for forest images. These datasets contributed to the Neither category.

**Figure 1:**
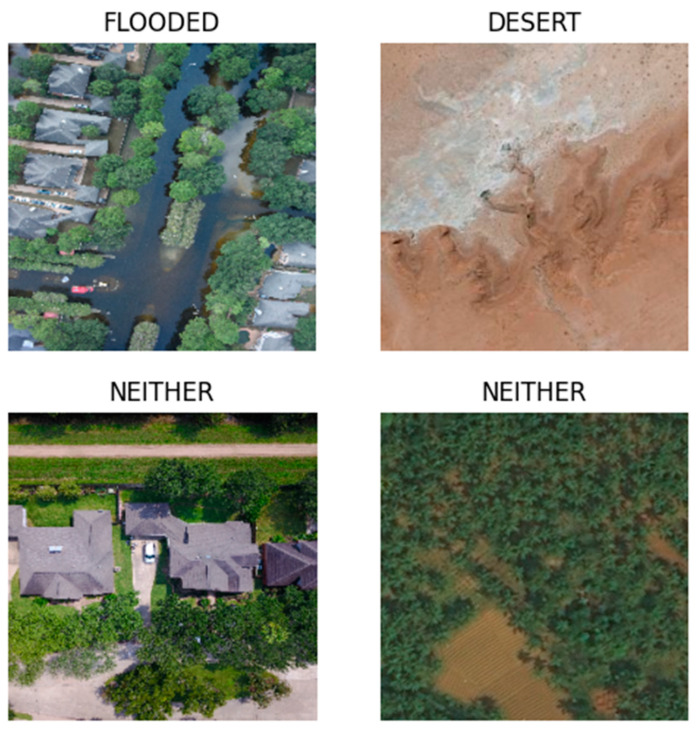
These are example images from the Climate Change Dataset. The example images on top show an example image from the Flood and Desert category. On the bottom row, the example images show the corresponding example image from the Neither category.

The image dimensions include variable sizes ranging from (224 × 224 pixels) to (over 3 K pixels × over 4 K pixels). Both jpeg and png image formats were used in the dataset. Representative images can be seen in Figure 1. To avoid an imbalance of classes, we strove for each category to contain 1/3 of the total images. Our goal was to find at least 6 K relevant images with 2 K in each of the three categories. Unfortunately, fewer images related to desertification were found. From preliminary results on a smaller dataset, we found that the ML models were very precise at identifying the desert category. For this reason, we allowed the desert category to contain slightly fewer images than the other categories.

### 2.2. Preprocessing and Model Initiation

Images from the Climate Change Dataset were screened and selected through a vigorous quality control process. Images were filtered to screen out: non-aerial images, logos, borders, and extremely blurry images. The datasets were manually perused and subjected to this quality control screening. Only images related to their appropriate category remained within the Climate Change Dataset.

During preprocessing, the images from the Climate Change Dataset were resized to 64 × 64 pixels to fit the CNN model we built. The 64 × 64 pixels image size was chosen for a faster ML run speed. Alternatively, for the transfer learning models, the images were resized to 224 × 224 pixels instead to fit those models. Next, the dataset was split into the training set (80%) and testing set (20%). The Climate Change Dataset, following these preprocessing steps, was then loaded into our 4 ML models.

### 2.3. VGG16 Network Model

**Figure 2:**
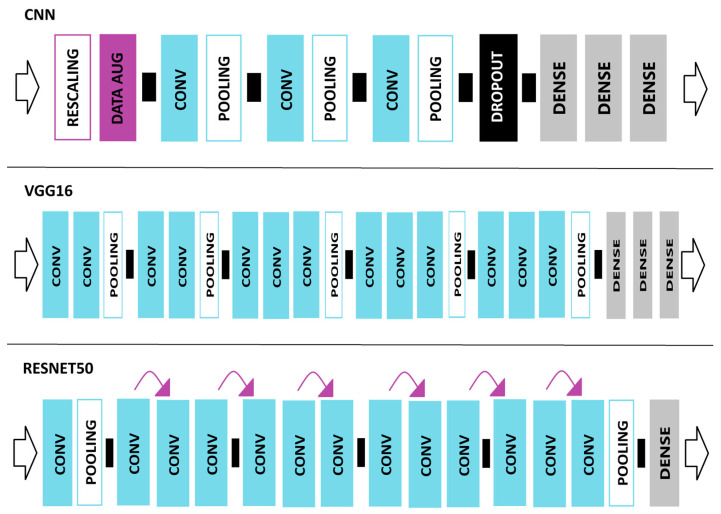
These diagrams represent the model architecture of 3 of the ML models used (CNN, VGG16, ResNet50). The left arrows represent input loading into the ML model. CONV stands for convolutional layers. POOLING stands for pooling layers (max, average, or global). DATA AUG stands for a Data Augmentation layer. The purple arrows represent skip connections. The right arrow represents the output of the ML model.

In this Section and in the next few Sections, we will discuss the 4 ML models utilized in our study. VGG16 [54] was chosen for its reputation as a high-performing ML model for image classification. VGG refers to the Visual Geometric Group from Oxford University where Simonyan and Zisserman developed the VGG network model [54]. The VGG-16 model included 3 fully connected layers and 13 convolutional layers. We used the VGG-16 model pre-trained on the ImageNet dataset [55]. The VGG network model became well known when the model received one of the top prizes for the ImageNet Large Scale Recognition Challenge in 2014 [56]. VGG [54] outperformed other ML models during the image classification challenge through its deep architecture of 16 to 19 weight layers with small (3 × 3) convolution filters [54]. The VGG16 [54] model architecture can be seen in Figure 2.

### 2.4. DenseNet201 Network Model

DenseNet [57] was recommended by a colleague in AI research for the ML model’s high testing accuracy. The DenseNet201 [57] network model is a Dense Convolutional network. The dense connections lead to higher testing accuracy when compared to other network models. Each layer of the DenseNet [57] model receives input from all preceding layers in a feed-forward fashion [57]. Features from the various layers of the DenseNet [57] model are concatenated together, which encourages the reuse of learned features. These characteristics of DenseNet’s [57] model architecture have led to DenseNet’s [57] outstanding performance [57]. DenseNet’s [57] dense connections alleviate the vanishing gradient problem, as well [57].

**Figure 3:**
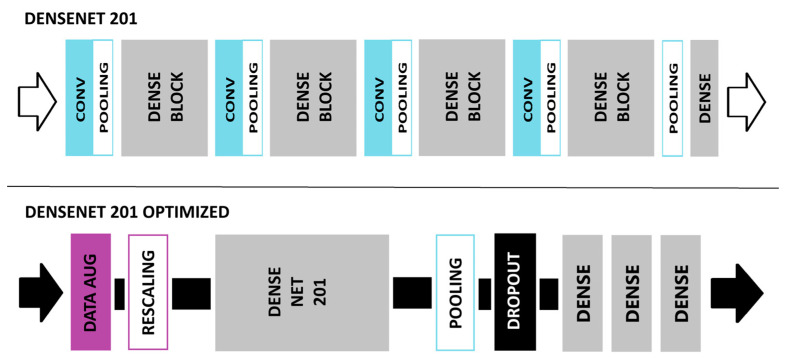
These diagrams represent the model architecture of 2 of the ML models used (DenseNet201 Basic and DenseNet201 Optimized). The left arrows represent input loading into the ML model. CONV stands for convolutional layers. POOLING stands for pooling layers (max, average, or global). DATA AUG stands for a Data Augmentation layer. DENSENET201 refers to the pre-trained version of the model. DENSE BLOCK represents several Dense layers with each layer receiving input from the preceding layer in a feed-forward fashion. The right arrow represents the output of the ML model.

DenseNet [57] was further optimized to increase the testing accuracy for natural disaster detection. Numerous layers were added to the model architecture. These layers are discussed below. The basic DenseNet201 [57] model architecture and the DenseNet201 Optimized [57] model architecture are shown in Figure 3.

#### 2.4.1. Data Augmentation Layer

Data augmentation increases the diversity of your dataset [58]. This Keras preprocessing experimental layer, called the data augmentation layer, adjusts images for better testing accuracy of new data. These adjustments increase the variety of images available from smaller datasets. Zoom, contrast, flip, and brightness are all examples of possible adjustments that can be made to the images in the dataset. Several random transformations are applied to the images to accomplish this task. Random contrast and random zoom transformations were applied to our dataset of images. Random contrast was set to 0.3 and random zoom was set to 0.1. The data augmentation layer is a helpful tool to increase testing accuracy [58].

#### 2.4.2. Rescaling Layer

A rescaling layer was added to the ML model architecture. The images were normalized by rescaling the original pixel values to values between 0 and 1. The empirical method was used, which divides the pixel intensity value by 255. Normalizing the pixel intensity through the empirical method yields higher testing accuracies for ML classification [59].

#### 2.4.3. Global Average Pooling Layer

A Global Average Pooling (GAP) layer was added to our DenseNet [57] model. The GAP layer takes the average of the feature map for each category. This layer is useful in avoiding overfitting [60].

#### 2.4.4. Dropout Layer

The overfitting of neural networks to training data is a serious problem that can be avoided by the addition of a dropout layer [61]. A dropout layer was added to reduce overfitting of our DenseNet [57] model to the data. A portion of neurons was excluded to promote variations in the data. A dropout rate of 0.2 was selected. The dropout layer enhances the capability of our DenseNet [57] model to generalize new, never seen, data [32].

#### 2.4.5. Fully Connected Layer and Classifier

The fully connected layer (FC) is a key, integral part of convolutional neural networks [62]. For shallow CNNs, the FC layer is required since the features found by the last convolutional layer of a shallow CNN do not cover the entire spatial image [62]. Instead, only a portion of the overall image is being represented in the feature map. For FC layers, each neuron in the layer is connected to each neuron in the previous layer enabling high-level feature extraction [32]. For our DenseNet [57] model, we used 3 FC layers containing 64, 64, and 3 neurons.

Located in the final layer, the SoftMax classifier is crucial for accurate image classification. The SoftMax classifier assigns a probability between 0 to 1 for each category, giving an indication of the confidence level of the model’s prediction [32]. The summation of probabilities totals 1.

### 2.5. ResNet50 Network Model

We utilized ResNet [63] in our Python model due to its history of outperforming in ML challenges. In 2015, the ResNet50 [63] network model received one of the top prizes in the ImageNet Large Scale Recognition Challenge. The ResNet50 [63] network model is a CNN containing 50 layers. The vanishing gradient problem leads to higher error rates in neural networks as the network becomes deeper [64]. This challenge was overcome with the skip connection technique incorporated into the ResNet50 [63] model. The skip connection creates a shortcut between layers in order to enable a direct connection to the output [64]. ResNet50 outperformed many other ML models in image classification problems by alleviating the vanishing gradient problem [63]. The ResNet50 [63] model architecture can be seen in Figure 2.

### 2.6. Transfer Learning Framework

**Figure 4:**
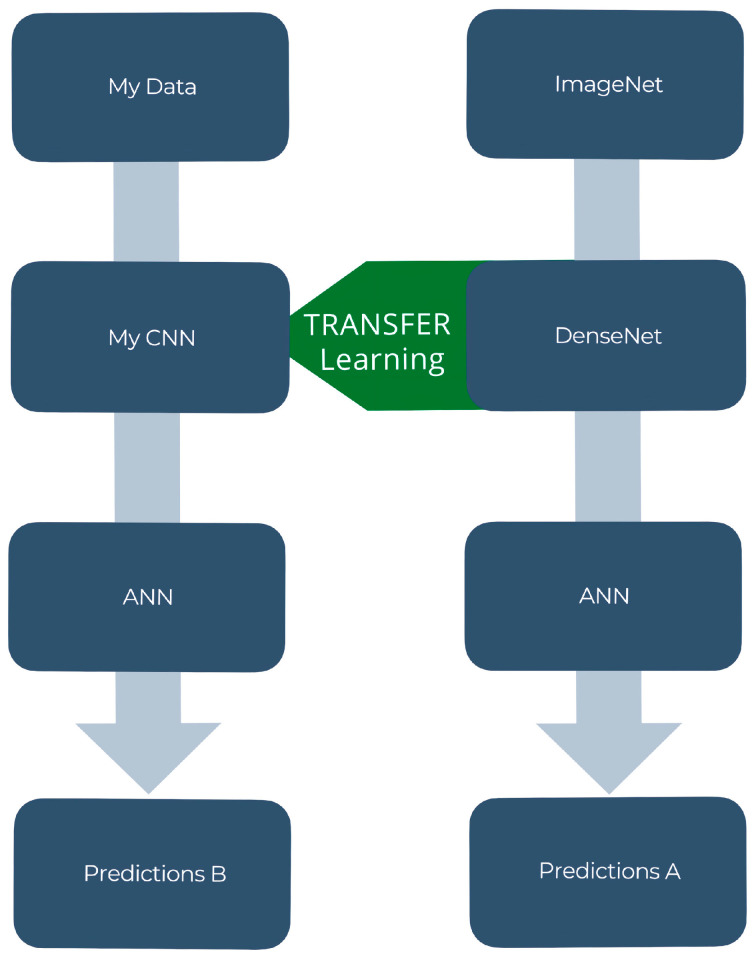
Transfer Learning Framework.

ML models (VGG16 [54], DenseNet201 [57], ResNet50 [63]) pre-trained on the ImageNet dataset [55] were utilized for our ML module. Features learned previously to make predictions were applied to this new problem of climate change-related natural disaster detection. The transfer learning framework is visualized in Figure 4. Transfer learning models have been well documented for yielding high testing accuracies on new datasets [33,51,56,64]. Our Climate Change Dataset was loaded into each of these pre-trained ML models. New predictions were then made utilizing transfer learning techniques. Three pre-trained ML models were compared: VGG16 [54], DenseNet201 [57], and ResNet50 [63]. All three transfer learning models were selected for their superior performance on image classification challenges. The top-performing ML model out of the numerous pre-trained transfer learning models being compared can often vary dependent on the dataset being used. For example, Abu et. al. found DenseNet to perform the best on their dataset [65] but Yang et. al. found ResNet to perform the best on their dataset [66].

### 2.7. Convolutional Neural Network (CNN) Model

We built our own rudimentary convolutional neural network (CNN) for the purpose of detecting climate change-related natural disasters. A detailed review of layers utilized in ML models is given in Section 2.4.

#### 2.7.1. CNN Layers

A quick mention of the layers utilized in our CNN model will be revealed in this Section. The CNN model we built contains the following:

- 1 rescaling layer;
- 1 data augmentation layer;
- 3 convolutional layers;
- 3 pooling layers;
- 1 drop-out layer;
- 3 fully connected (FC) layers.

The final layer utilizes a SoftMax classifier for image classification. The rescaling layer normalized the image data from the original pixel intensity values into values between 0 and 1. The empirical method was used when the original pixel intensity values were divided by 255. In the data augmentation layer, we selected random contrast at 0.3 and random zoom at 0.1. The convolutional layers took an initial input of 64 × 64 × 3 with ReLU activation. Detailed information on the pooling layers is given below. For the drop-out layer, we selected 0.4 to reduce overfitting and to improve testing accuracy. The three FC layers contained 32, 64, and 3 neurons each. Our CNN model architecture can be seen in Figure 2.

#### 2.7.2. Pooling Layers

Three pooling layers were added to our CNN model. Average pooling was used for the first pooling layer. Average pooling calculates the average value for the given region [67]. Max pooling was used for the last two pooling layers. Max pooling selects the largest value for the given region. Both average pooling and max pooling are feature extraction techniques. The pooling layer takes the feature map from the previous layer and pools the data from small local regions to build a new feature map with reduced spatial dimension [67].

### 2.8. Experimental Setup

This Section describes the hardware and software environment used to run the experiments with different ML Python modules. The running of Transfer Learning modules is memory intensive. For this reason, our ML module was run with Google Colab Tensor Processing Unit (TPU) high-RAM v2-8 cloud. Google Colab was used for the following:

- Collaborate online with code/feedback;
- Accelerate our ML workload with Google GPUs/TPUs;
- Utilize Google’s cloud computing resources.

The code was written in Python version 3.11.1. TensorFlow, Keras, and Scikit-Learn ML libraries were installed and utilized throughout our ML module.

### 2.9. Evaluation Metrics

In this Section, we explain the evaluation metrics used to measure the performance of our ML models while providing the pertaining formulas, as well [38]. We utilized the following metrics: Accuracy, Confusion Matrix, Precision, Recall, and F1-Score. The Confusion Matrix was chosen to visually display the performance of our ML models. The predicted and actual classifications were compared within the confusion matrices. These tables show true positives (*TP*), true negatives (*TN*), false positives (*FP*), and false negatives (*FN*). Accuracy calculates the fraction of correct predictions per total predictions made by the ML model. The accuracy formula is given in Equation (1).

(1) $$Accuracy=\frac{TP+TN}{TP+TN+FP+FN}$$

Recall, also known as sensitivity, calculates the fraction of positives predicted correctly by the ML model per total actual positives in the batch. The Recall measures the ML model’s capability of correctly identifying the total actual positive cases. The Recall formula is expressed in Equation (2).

(2) $$Recall=\frac{TP}{TP+FN}$$

Precision calculates the fraction of true positives per total positives predicted by the ML model. Precision is a measure of the quality of the ML model’s positive predictions. The Precision formula is given in Equation (3).

(3) $$Precision=\frac{TP}{TP+FP}$$

The F1 score measures an ML model’s performance based on the precision and recall values. This metric calculates the harmonic mean of the precision and recall values [38]. The F1 Score formula is provided in Equation (4).

(4) $$F1\;Score=2*\frac{Precision*Recall}{Precision+Recall}$$

### 2.10. Cross-Validation Methods

For cross-validation purposes, one data subset was chosen to be removed from the larger full dataset. The first dataset left out was the Aerial Landscape Images [49] dataset from the training set. Altogether, two datasets were utilized for this technique. The second dataset contained the data subset [49]. For cross-validation purposes, our Python module was run with a testing set containing the full dataset including the data subset [49], even though, the ML model was not trained on that particular subset of data. We utilized a cross-validation technique related to the Leave-One-Out Cross-Validation dataset method [68].

## 3. Results

In this Section, we provided our experimental results. We began by displaying the individual performance of our four ML models (VGG16 [54], CNN, ResNet50 [63], and DenseNet201 [57]) for climate change-related natural disaster detection. A comparison of the four ML models was made. Optimization techniques were discussed. We demonstrate how performance was improved with optimization. Finally, we juxtaposed two of our highest-performing ML models, ResNet50 [63], and DenseNet201 [57]. Testing accuracies and testing loss were plotted for all four ML models. The testing set was used for validation purposes. The cross-entropy loss was calculated for the validation loss graphs.

### 3.1. Individual Model Performance

We demonstrate the performance of each single ML model by visually displaying each ML model’s testing accuracy, testing loss, and confusion matrix across 70 epochs. We used a batch size of 64.

#### 3.1.1. VGG16 Performance

**Figure 5:**
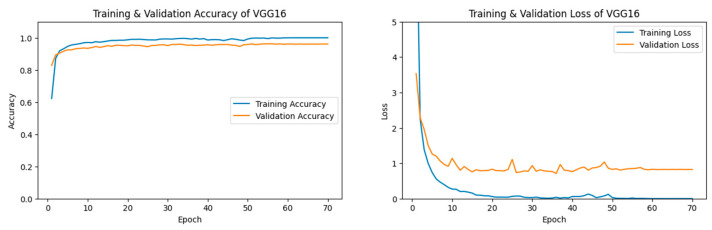
The left figure shows the accuracy curve for our VGG16 model. The right figure shows the loss curve for our VGG16 model.

Our VGG16 [54] model displayed a relatively high validation accuracy. A final validation accuracy of 96.13% was reached after 70 epochs, as seen in Figure 5.

**Figure 6:**
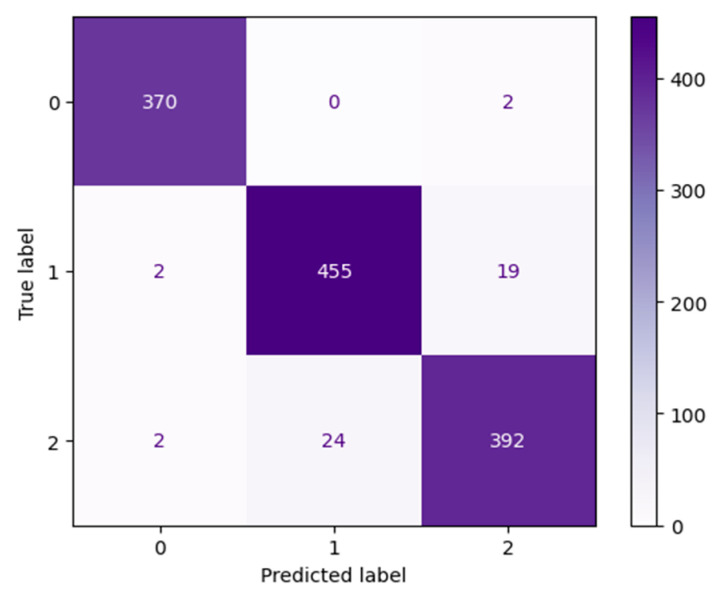
Confusion matrix for VGG16 after 70 epochs.

The confusion matrix for VGG16 [54] can be seen in Figure 6. Label 0, the Desert category, showed the highest precision among categories. Less than 4% of the time, when an image from our Climate Change Dataset was inaccurately predicted, then that image was most likely from the Neither category, label 2.

In Table 2, the Desert category demonstrated an extremely high precision, recall, and F1-Score of 0.99. The Neither category had a lower recall and F1-Score of 0.94.

#### 3.1.2. DenseNet Performance

**Figure 7:**
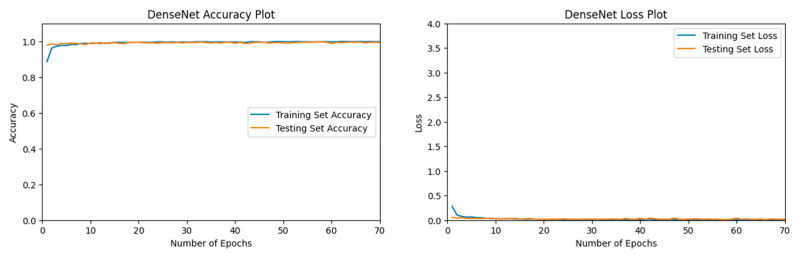
The left figure shows the accuracy curve for our DenseNet201 Optimized model. The right figure shows the loss curve for our DenseNet201 Optimized model.

Our DenseNet201 [57] Optimized model displayed extremely high validation accuracy of over 97% at epoch 1. The extremely low testing loss can be seen in Figure 7, as well. Our DenseNet201 [57] Optimized model reached 99.45% validation accuracy after 70 epochs.

**Figure 8:**
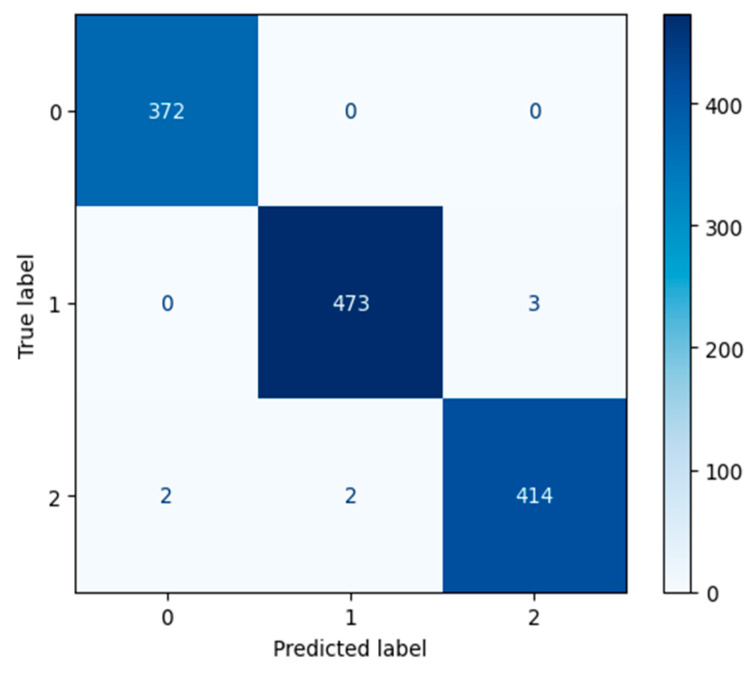
DenseNet201 Optimized model after 70 epochs.

All actual occurrences of label 0, the Desert Category, were accurately predicted by our DenseNet201 [57] Optimized model. As can be seen in the confusion matrix shown in Figure 8. Less than 1% of the time, when an image was predicted incorrectly, the image belonged to the Neither category, label 2.

The flooded category has one of the highest precision scores of 1.0 when classified by our DenseNet201 [57] Optimized model. Table 3 reveals the extremely high overall precision, recall, and F1-Scores for our DenseNet201 [57] Optimized model.

#### 3.1.3. CNN Performance

**Figure 9:**
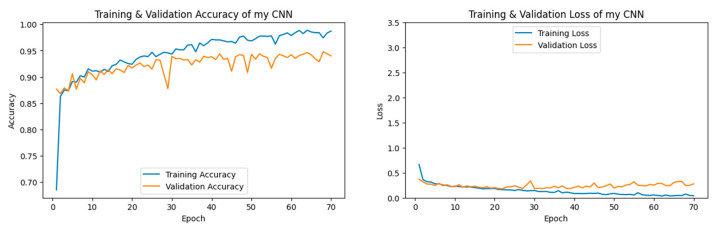
The left figure shows the accuracy curve for our CNN model. The right figure shows the loss curve for our CNN model.

Our CNN model was trained on our Climate Change Dataset. As can be seen in Figure 9, our CNN model displayed 94.00% accuracy after 70 epochs.

**Figure 10:**
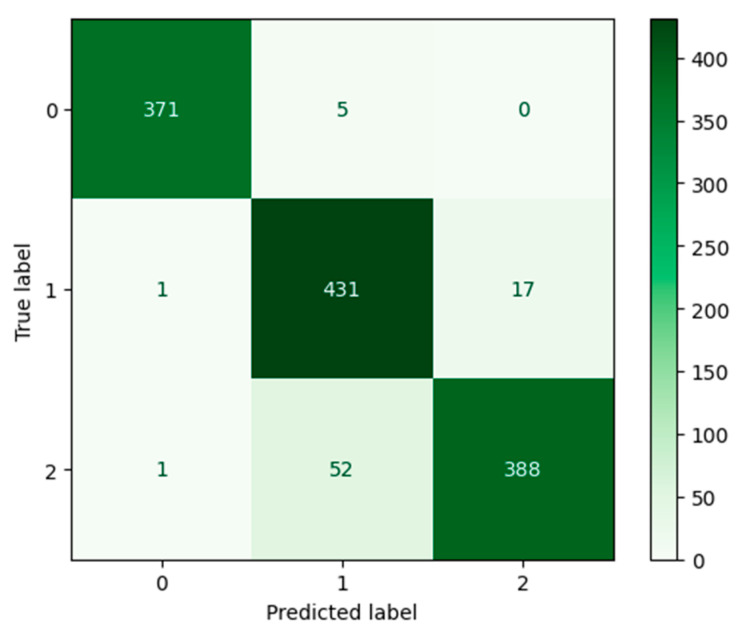
Confusion matrix for our CNN model after 70 epochs.

The Desert category, label 0, was predicted by our CNN model with high accuracy, according to the confusion matrix seen in Figure 10. Six percent of the time, when an image from our dataset was incorrectly predicted, then that image belonged to the Neither category, label 2.

According to Table 4, when compared to other categories, our CNN model demonstrated the highest scores for the Desert category with precision, recall, and F1-score reaching 0.99. The Neither category demonstrated a lower score of 0.88 on recall.

#### 3.1.4. ResNet Performance

**Figure 11:**
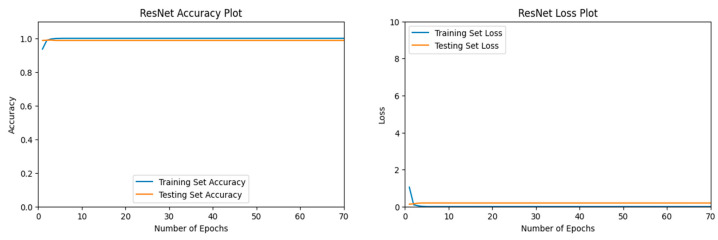
The left figure shows the accuracy curve for our ResNet50 model. The right figure shows the loss curve for our ResNet50 model.

Our ResNet50 [63] model reached validation accuracies of nearly 100%, as demonstrated in Figure 11. The final validation accuracy after 70 epochs was 98.74% on a separate run.

**Figure 12:**
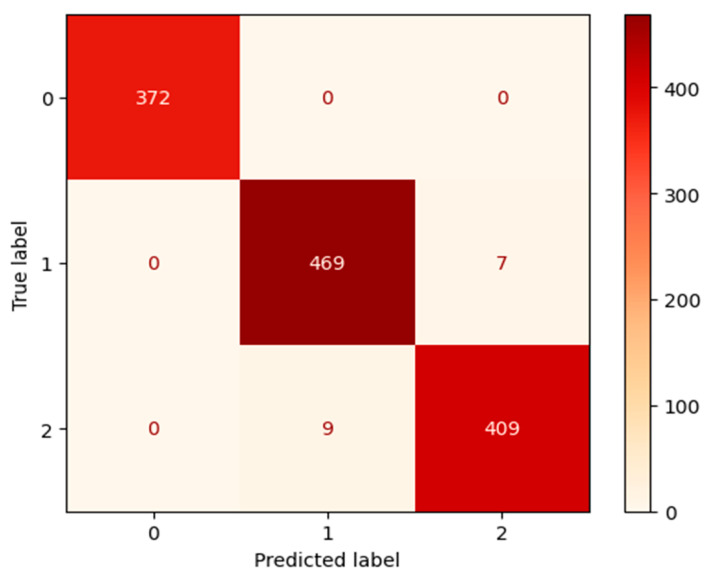
Confusion matrix for our ResNet50 model after 70 epochs.

The Desert category, label 0, was predicted accurately upon each actual occurrence and every prediction for the desert category was accurate, as well. The confusion matrix in Figure 12 shows the flawless predictions by our ResNet50 [63] model for the Desert category.

Table 5 confirms that the Desert category has a score of 1.0 for precision, recall, and F1-Score by our ResNet50 [63] model. The Neither category received a score of 0.98 for precision, recall, and F1-Score.

### 3.2. ML Model Comparison

**Figure 13:**
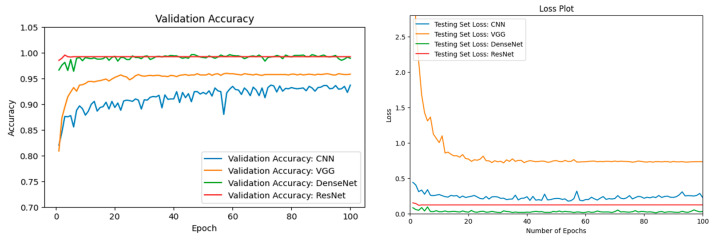
The left figure shows the accuracy curve for all 4 of our ML models (CNN, VGG16, DenseNet201 Optimized, ResNet50). The right figure shows the loss curve for all 4 ML models.

In this Section, we compare and contrast the performance of our four ML models for the task of climate change-related natural disaster detection. Our ML Python Module was run over 100 epochs with a batch size of 128. The testing accuracies and testing loss can be seen in Figure 13.

As seen in Table 6, after 100 epochs, our ResNet50 [63] model and our DenseNet201 [57] Optimized model reached similar high validation accuracies of 99.21% and 98.89% respectively. Our VGG16 [54] model converged to a validation accuracy between our CNN model and our DenseNet201 Optimized [57] model.

### 3.3. Optimization of DenseNet

**Figure 14:**
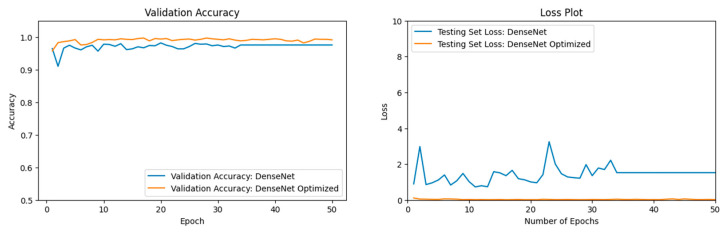
The left figure shows the accuracy curve for our basic DenseNet201 model vs. our DenseNet201 Optimized model. The right figure shows the loss curve for our basic DenseNet201 model vs. our DenseNet201 Optimized model.

Our DenseNet201 [57] model performed well during preliminary results from a smaller dataset and was, therefore, chosen for optimization. The DenseNet201 [57] Optimized model contained additional layers such as a Rescaling layer and a Data Augmentation layer. The two ML models, our basic DenseNet201 [57] and our DenseNet201 [57] Optimized, were run over 50 epochs with a batch size of 64. The performance of both our basic DenseNet201 [57] model and our DenseNet201 [57] Optimized model can be seen in Figure 14. The basic DenseNet201 [57] model, across epochs, converged to a validation accuracy lower than the optimized DenseNet201 [57] model. The optimized model with Rescaling and Data Augmentation layers has yielded consistently higher validation accuracies. The validation loss is much lower for the optimized model, as well.

According to Table 7, our DenseNet201 [57] Optimized model yielded a higher validation accuracy than our basic DenseNet201 [57] model.

### 3.4. ResNet vs. DenseNet Optimized

**Figure 15:**
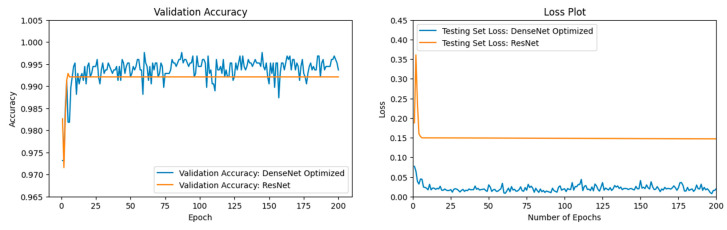
The left figure shows the accuracy curve for our DenseNet201 Optimized model vs. our ResNet50 model. The right figure shows the loss curve for our DenseNet201 Optimized model vs. our ResNet50 model.

In our previous Section 3.2, the ResNet50 [63] model and the DenseNet201 [57] Optimized model appeared to perform similarly on the validation accuracy plot. To more clearly visualize their performance, our ResNet50 [63] model was juxtaposed with our DenseNet201 [57] Optimized model for a longer run over more epochs. Our ML Python module was run for 200 epochs with a batch size of 128. The validation accuracies for our DenseNet201 [57] Optimized model oscillated from approximately 0.987–0.998, as can be seen in Figure 15. DenseNet201 [57] Optimized demonstrated a lower testing loss and a consistently higher testing accuracy when running 200 epochs. The ResNet50 [63] model converged to a validation accuracy lower than the DenseNet201 Optimized [57] model’s validation accuracy over the vast majority of the epochs.

As can be seen in Table 8, our DenseNet201 [57] Optimized model reached a higher validation accuracy of 99.37% after 200 epochs.

### 3.5. Cross-Validation

In this Section, we cross-validate the performance of our four ML models for the task of climate change-related natural disaster detection. Our ML Python Module was run over 100 epochs with a batch size of 128. As can be seen in Table 9, the validation accuracies for all four ML models remained high, with validation accuracies over 95%.

## 4. Discussion

Our compiled Climate Change Dataset provided enough images for our ML models to extract key features in accurately detecting the natural disasters examined in this study. Our four ML models all performed well at climate change-related natural disaster detection based on images from our Climate Change Dataset. The four ML models, VGG16, CNN, DenseNet201 Optimized, and ResNet50, all reached high validation accuracies of 95.81%, 93.68%, 98.89%, and 99.21%, respectively, over 100 epochs. The categories in our dataset were easy for our ML models to distinguish.

Our DenseNet201 model was selected for optimization techniques such as data augmentation. DenseNet201 was selected due to its superior performance in preliminary results from a smaller dataset. Validation accuracy increased from 97.55% to 99.13% when additional layers were added to DenseNet201 for optimization purposes. The testing set loss was lower, as well, with cross-entropy loss dropping from 1.5234 to a much lower cross-entropy loss of 0.0196. Adding a Dropout layer, a Rescaling layer, and a Data Augmentation layer improved the performance of our DenseNet201 model. Rescaling layers and Data Augmentation layers have been shown to increase testing accuracies [58,59]. Both Dropout layers and Data Augmentation layers help prevent overfitting of ML models [58,61].

Although all four ML models performed well at natural disaster detection, ResNet50 and DenseNet201 Optimized yielded higher validation accuracies and appeared to demonstrate similar performance on the validation accuracy plot (Figure 15). Both ML models have a model architecture that alleviates the vanishing gradient problem of deeper models [57,63]. Oluibukun Gbenga Ajayi and John Ashi found that the ML model validation accuracy showed an increasing upward trend as the number of epochs increases until the validation accuracy finally converges [69]. We ran the two ML models over a higher epoch count to compare the two ML models. ResNet50 and DenseNet201 Optimized reached 99.21% and 99.37%, respectively, over 200 epochs. From Figure 15, the DenseNet201 Optimized model consistently yielded higher testing accuracy than the ResNet50 model over the span of 200 epochs the vast majority of the time. Still, the difference between the two ML models’ testing accuracy was slight. For ML professionals that prefer validation accuracies in the 90–97% range, then our VGG16 and our CNN models would suit their purposes better. Our CNN needed to find the important features and ignore any noise in the data for accurate DL detection. Therefore, the learning process was gradual for our CNN.

High testing accuracy at the beginning could mean that our other ML models were overconfident. We were concerned about overfitting occurring from the beginning of our project. Overfitting can be detected in the loss curves [70]. In the case of overfitting, the validation loss curve would increase and be much higher than the training loss curve [70]. Our loss curves decline gradually across epochs.

We cross-validated our results. We utilized a cross-validation technique related to the Leave-One-Out Cross-Validation dataset method [68]. Testing accuracies were still relatively high. All four ML models obtained a final testing accuracy of over 95%.

For future improvements, more images could be added to our dataset. More categories could be added, as well. Wildfires could be added to the list of climate change-related natural disaster categories. To further our research project, our Python module could be applied to drone emergency response. After the natural disaster is detected, survivors could be located by the UAV. Aid could then be rendered by programming the UAV to provide life-saver safety vests to the survivors in the case of a flood. Our study was limited by the reliance upon the amount of data within our dataset and by the data source. Our project was also limited in the number of natural disaster types considered for our study.

Our study highlights the power of AI in addressing some of the most pressing environmental challenges. This project demonstrated how interdisciplinary efforts can be integrated to find comprehensive solutions to global problems. Our study intended to use AI for Climate Change and Environmental Sustainability. We are determined to use AI for the benefit of society, particularly in mitigating the negative effects of climate change.

The Python module is available upon request. Contact the corresponding author.

## Figures and Tables

**Table 1:** Climate Change Dataset Image Counts.

Name of Dataset	Total Image Count	Flooded	Desert	Neither
Louisiana Flood 2016 [44]	263	102	0	161
FDL_UAV_flood areas [45]	297	130	0	167
Cyclone, Wildfire, Flood, Earthquake Database [46]	613	613	0	0
Satellite Image Classification Disaster Dataset [47]	1131	0	1131	0
Disasters Dataset [48]	1630	1493	0	137
Aerial Landscape Images [49]	800	0	800	0
Aerial Images of Cities [50]	600	0	0	600
Forest Aerial Images for Segmentation [51]	1000	0	0	1000
Totals	6334	2338	1931	2065

**Table 2:** Evaluation of our VGG16 model image classification results.

Category	Precision	Recall	F1-Score
Desert	0.99	0.99	0.99
Flooded	0.95	0.96	0.95
Neither	0.95	0.94	0.94

**Table 3:** Evaluation of our DenseNet201 Optimized model image classification results.

Category	Precision	Recall	F1-Score
Desert	0.99	1.0	1.0
Flooded	1.0	0.99	0.99
Neither	0.99	0.99	0.99

**Table 4:** Evaluation of our CNN model image classification results.

Category	Precision	Recall	F1-Score
Desert	0.99	0.99	0.99
Flooded	0.88	0.96	0.92
Neither	0.96	0.88	0.92

**Table 5:** Evaluation of our ResNet50 model image classification results.

Category	Precision	Recall	F1-Score
Desert	1.00	1.00	1.00
Flooded	0.98	0.99	0.98
Neither	0.98	0.98	0.98

**Table 6:** Validation accuracy of our 4 ML models.

ML Model	Validation Accuracy
CNN	0.9368
VGG16 [54]	0.9581
DenseNet201 [57] Optimized	0.9889
ResNet50 [63]	0.9921

**Table 7:** Validation accuracy of our DenseNet201 model before and after optimization.

ML Model	Validation Accuracy	Validation Loss
DenseNet201 [57]	0.9755	1.5234
DenseNet201 [57] Optimized	0.9913	0.0196

**Table 8:** Validation accuracy of our ResNet50 model and our DenseNet201 Optimized model.

ML Model	Validation Accuracy
ResNet50 [63]	0.9921
DenseNet201 [57] Optimized	0.9937

**Table 9:** Cross-Validation accuracy of our 4 ML models.

ML Model	Validation Accuracy
CNN	0.96
VGG16 [54]	0.97
DenseNet201 [57] Optimized	0.96
ResNet50 [63]	0.97

## Data Availability

Data available online for peer review (doi: 10.5281/zenodo.14397148).
